# Neuromuscular Electrical Stimulation of the Quadriceps in Patients with Non-Small Cell Lung Cancer Receiving Palliative Chemotherapy: A Randomized Phase II Study

**DOI:** 10.1371/journal.pone.0086059

**Published:** 2013-12-30

**Authors:** Matthew Maddocks, Vanessa Halliday, Alpna Chauhan, Victoria Taylor, Annmarie Nelson, Cathy Sampson, Anthony Byrne, Gareth Griffiths, Andrew Wilcock

**Affiliations:** 1 Kings College London, Cicely Saunders Institute, Department of Palliative Care, Policy & Rehabilitation, London, United Kingdom; 2 Department of Palliative Medicine, Nottingham University Hospitals National Health Service Trust, Nottingham, United Kingdom; 3 School of Biosciences, University of Nottingham, Nottingham, United Kingdom; 4 King’s Mill Hospital, Sherwood Forest Hospitals National Health Service Foundation Trust, Sutton in Ashfield, United Kingdom; 5 Marie Curie Research Centre and Wales Cancer Trials Unit, Cardiff University, Cardiff, United Kingdom; Universidad Europea de Madrid, Spain

## Abstract

**Background:**

A reduced exercise capacity is associated with increased morbidity and mortality in patients with advanced non-small cell lung cancer (NSCLC). Therapeutic exercise can be beneficial and neuromuscular electrical stimulation (NMES) of the quadriceps muscles may represent a practical approach. The primary aim of this study was to determine the acceptability of NMES of the quadriceps to patients with NSCLC used alongside palliative chemotherapy. Secondary aims explored aspects of safety and efficacy of NMES in this setting.

**Methods:**

Patients with advanced NSCLC due to receive first-line palliative chemotherapy were randomized to usual care with or without NMES. They were asked to undertake 30 minute sessions of NMES, ideally daily, but as a minimum, three times weekly. For NMES to be considered acceptable, it was predetermined that ≥80% of patients should achieve this minimum level of adherence. Qualitative interviews were held with a subset of patients to explore factors influencing adherence. Safety was assessed according to the Common Terminology Criteria for Adverse Events. Quadriceps muscle strength, thigh lean mass, and physical activity level were assessed at baseline and after three cycles of chemotherapy.

**Results:**

49 patients (28 male, median (IQR) age 69 (64−75) years) participated. Of 30 randomized to NMES, 18 were eligible for the primary endpoint, of whom 9 (50% [90% CI, 29 to 71]) met the minimum level of adherence. Adherence was enhanced by incorporating sessions into a daily routine and hindered by undesirable effects of chemotherapy. There were no serious adverse events related to NMES, nor significant differences in quadriceps muscle strength, thigh lean mass or physical activity level between groups.

**Conclusions:**

NMES is not acceptable in this setting, nor was there a suggestion of benefit. The need remains to explore NMES in patients with cancer in other settings.

**Trial Registration:**

Current Controlled Trials ISRCTN 42944026 www.controlled-trials.com/ISRCTN42944026

## Introduction

In patients with advanced non-small cell lung cancer (NSCLC), a reduced exercise capacity is associated with increased morbidity and mortality [1,2]. Causative factors include the cancer, via the cachexia syndrome [3,4], and cancer treatments, with patients receiving palliative chemotherapy experiencing an overall deterioration in muscle strength, lean body mass and physical activity level [5,6]. In part, these effects arise from chemotherapy-related fatigue contributing towards a negative cycle of reduced physical activity and muscle deconditioning [7].

Therapeutic exercise may help mitigate these effects [1,8] and exercise programmes with aerobic and/or resistance training have been explored in patients with advanced NSCLC [9-11]. However, limitations include relatively low rates of uptake and adherence, and inconsistent benefit, which may be limited to select well motivated patients [9,11].

Neuromuscular electrical stimulation (NMES) of the lower limb muscles is a form of exercise undertaken whilst seated, which requires less motivation and change in lifestyle than traditional exercise. It could provide a pragmatic alternative, particularly for those patients experiencing exercise-limiting symptoms, e.g. breathlessness. Further, patients with advanced cancer have expressed a general preference for NMES over traditional forms of exercise [12]. Although NMES offers no aerobic training, improvements in quadriceps strength and exercise capacity have been achieved in patients with advanced disease [13,14]. A pilot study undertaken by our group in patients with advanced NSCLC who had completed chemotherapy, suggested NMES warranted further study [15]. However, given the deleterious effects of palliative chemotherapy, we considered it appropriate to formally explore the acceptability of NMES in this setting before attempting a large confirmatory study. Thus, in conjunction with the UK National Cancer Research Institute Palliative Care Clinical Studies Group we developed a phase II trial with the primary aim to determine if NMES is an acceptable exercise intervention for patients with NSCLC receiving first-line palliative chemotherapy. Secondary aims were to examine aspects of safety and efficacy of NMES.

## Materials and Methods

The protocol for this trial and supporting CONSORT checklist are available as supporting information; see Checklist S1 and Protocol S1.

### Participants

**Figure 1:**
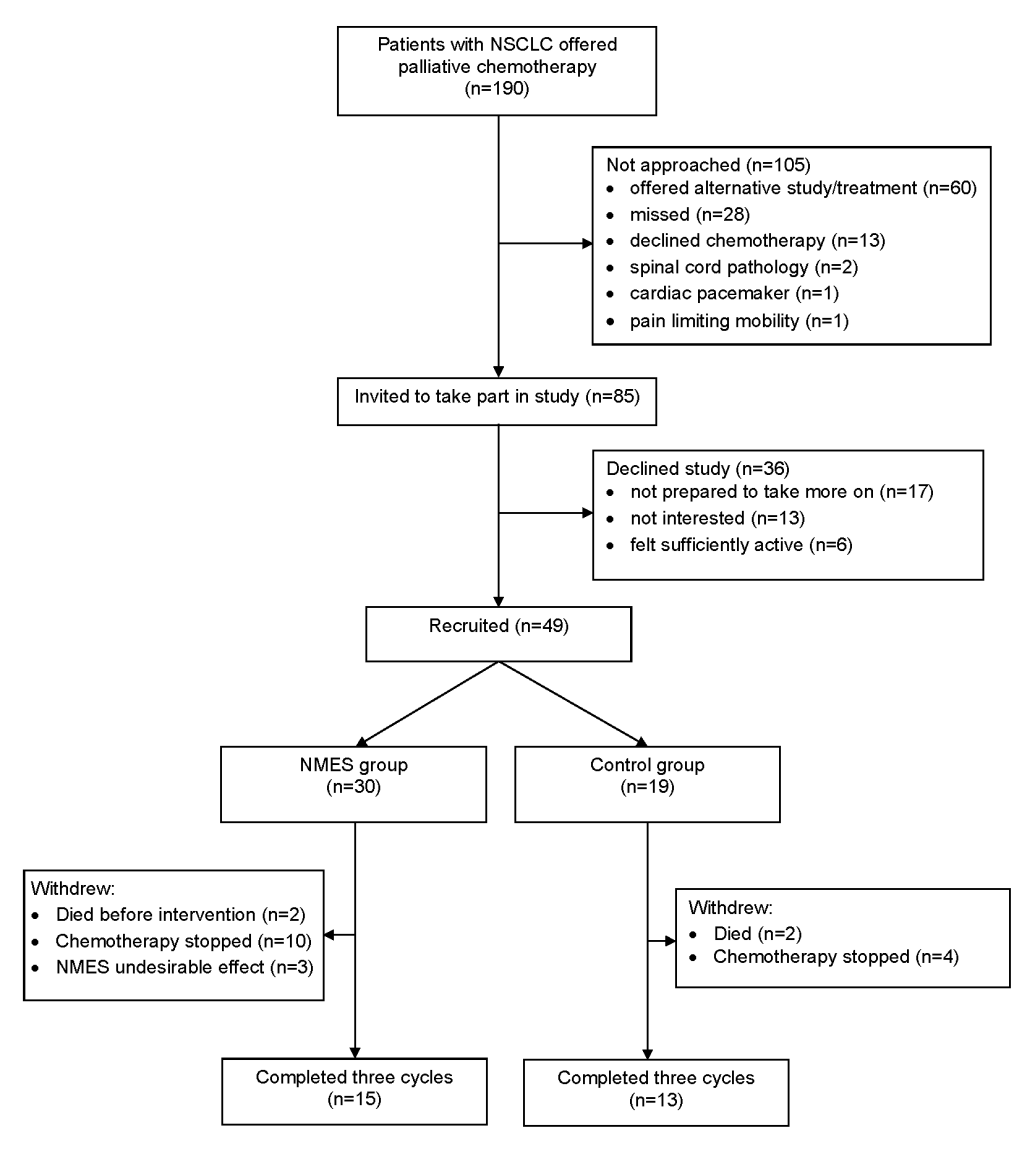
Study flow diagram

Eligible patients were identified from thoracic oncology clinics at a large teaching hospital (Nottingham University Hospitals NHS Trust) and a smaller neighbouring hospital (Sherwood Forest Hospitals NHS Foundation Trust) between September 2010 - May 2012 and September 2011 – May 2012 respectively (Figure 1; study flowchart). Inclusion criteria were: adults with advanced (stage IV) NSCLC confirmed by histology or cytology, an Eastern Cooperative Oncology Group (ECOG) performance status of 0–2, and scheduled to receive first-line palliative chemotherapy. Exclusion criteria were spinal cord compression, epilepsy or cardiac pacemaker. Participants gave written informed consent and the study was approved by Nottingham 1 Research Ethics Committee (ref. 09/H0403/24) and registered with Current Controlled Trials (ISRCTN 42944026).

### Procedures

Patients were randomized by the Wales Cancer Trials Unit using permuted blocks 1:1 into NMES or control (no NMES) groups. In September 2011, because of slower than anticipated recruitment, this ratio was amended to 2:1 in favour of NMES, along with the opening of a second recruiting site. Stratification factors were age (≤/>65), gender and concurrent use of long-term oral corticosteroids. Assessments took place before chemotherapy commenced and repeated during the third week of the third cycle of chemotherapy (i.e. after 9 weeks). Assessments were carried out at the hospital except for physical activity level, which was assessed under free-living conditions over the week prior to the hospital visit. Outcome assessors were not blinded to the patient group allocation.

### Neuromuscular electrical stimulation

Delivered by a MicroStim Exercise Stimulator MS2v2 (Odstock Medical Ltd, Wiltshire, UK) with two 7cm round PALS^®^ Platinum self-adhesive electrodes (Axelgaard Manufacturing Co Ltd, Denmark) placed on the anterior thigh over the quadriceps. Stimulation parameters were chosen to minimise fatigue and skin irritation whilst producing meaningful muscle contraction: (i) symmetrical biphasic squared pulses at 50Hz frequency, (ii) 350 microsecond pulse width, (iii) duty cycle increasing on a weekly basis from 11% to 18% to 25% and remaining constant thereafter [16]. The amplitude (device output 0–120mA, tested across 1000Ω) was initially set to elicit a visible and comfortable muscle contraction and patients were encouraged to increase the amplitude as tolerated. The research team supervised the first training session, offered written instruction and made contact weekly by telephone to provide feedback and adjust stimulation parameters. Patients were instructed to undertake bilateral thigh stimulation for 30 minutes, ideally daily, but as a minimum three times a week. This began one week after commencement of chemotherapy and continued for 8 or 11 weeks, dependent on patients receiving 3 or 4 cycles of chemotherapy respectively.

### Assessments

#### Acceptability

Assessed by patients' adherence to the recommended programme of NMES through self-report daily diaries over the initial 8 week period. At the end of the NMES intervention, a subset of patients was invited to complete semi-structured interviews to explore factors influencing adherence using inductive thematic analysis [17].

#### Quadriceps muscle strength

Assessed using a Manual Muscle Tester dynamometer (Lafayette Instruments, USA) mounted on a custom-built rig. Patients were seated with hips and knees at 90° and the dynamometer placed just above the ankle of the dominant leg. Patients pushed as hard as possible against the dynamometer for three seconds, whilst the assessor prevented any movement to ensure an isometric contraction. Following a familiarisation test, patients undertook three attempts with standardised verbal encouragement and the peak force (kilograms, kg) maintained for over half a second was recorded.

#### Body composition

thigh and whole body lean tissue mass were determined using dual-energy X-ray absorptiometry (LUNAR Prodigy Advanced, GE Lunar, Madison, USA) with enCORE software (Version 13.6) using standard imaging and positioning protocols [18].

#### Physical activity level

Mean daily step count and time spent in an upright posture, i.e. standing or walking, were measured over six days using an ActivPAL™ accelerometer (PAL technologies Ltd, Glasgow, UK). This is worn on the mid-thigh continuously except for when showering or bathing. It is accurate over a wide range of walking speeds and daily activity and not falsely triggered by travel in a motor vehicle [19-21].

#### Nutritional intake

Mean daily protein (g/d) and energy (kcal/d) intake were calculated using NetWISP (V3.0, Tinuviel Software, UK) based on a prospective 3-day diary which included one weekend and two week days.

#### Fatigue

Assessed using the Multidimensional Fatigue Inventory (MFI-20), a 20-item scale with five domains (general, physical and mental fatigue, reduced activity, and reduced motivation) [22,23]. A change in subscale score of 3−4 is considered clinically important [24].

#### Quality of life

Assessed using the multidimensional European Organisation for the Research and Treatment of Cancer Quality of Life Questionnaire Core 30 (EORTC QLQ-C30) and the lung cancer module (LC-13) [25,26]. A change in subscale score of ≥10 is considered clinically important [27].

#### Safety relating to NMES

Assessments were reported in real time according to the Common Terminology Criteria for Adverse Events (version 4.0) of the National Cancer Institute [28].

#### Response to chemotherapy

Evaluated by Response Evaluation Criteria in Solid Tumors (RECIST) guidelines version 1.1 [29].

### Statistical analysis

The primary endpoint for acceptability was the proportion of patients in the NMES group reaching the predetermined minimum level of adherence to the recommended programme, i.e. three times every week over an 8 week period. Following advice from the National Cancer Research Institute Palliative Care Clinical Studies Group, we determined *a priori* that ≥80% of patients should adhere to this minimum level in order to warrant a phase III study in this setting and if ≤50% adhered, this would give grounds to reject further study. Using a Fleming's single-stage method (p1=0.5; p2=0.8; α=0·05; 90% power), we needed to recruit 20 patients in the NMES group and if ≥15 out of 20 patients adhere, this would warrant further investigation [30]. Participants discontinuing the intervention or withdrawing from the study because of a NMES-related undesirable effect were considered in the analysis but classified as non-adherent. Continuous data were expressed as median and inter-quartile range [IQR]. Patient characteristics were compared between control and NMES groups using the Wilcoxon rank sum test or exact Pearson chi-square test. Categorical data were expressed as proportions. Changes in secondary outcomes were compared between groups by mean differences with 95% confidence intervals in an exploratory per protocol analysis. All calculations were performed using Stata version 11. P values <0.05 were regarded as statistically significant.

## Results

### Participants

Of 85 eligible patients approached, 49 were recruited. Fifteen (50%) of 30 patients randomised to the NMES group and 13 (68%) of 19 patients in the control group completed three cycles of chemotherapy (Figure 1). Assessments were completed in full except for body composition, missing in eight patients due to inability to obtain a scan before chemotherapy started (n=5) or because of patient choice (n=3); a self-report diary was not returned in one patient who deteriorated and died. Participants (28 male) had a median (IQR) age of 69 (64−75) years, body mass index of 25.3 (21.5−28.1) kg/m^2^ and percentage weight loss from a pre-illness stable baseline of 1 (0−1) %. Twenty four (49%) met criteria for cancer cachexia based on one or more of: low appendicular skeletal muscle index (n=17); >5% weight loss (n=13), or body mass index <20kg/m^2^ (n=4)^31^. Chemotherapy regimens were platinum-based (carboplatin or cisplatin) generally combined with either vinorelbine or pemetrexed. As of 1st October 2012, 33 patients have died with a median [IQR] survival of 23 [19−31] weeks. There were no significant baseline differences between NMES and control groups, except that a higher proportion of patients in the control group met the criteria for cachexia (63% vs. 40%, p=0.01) (Table 1).

**Table 1:** Patient characteristics.

	*NMES (n=30*)	*Control (n=19)*
*Sex* (m / f)	16 / 14	12 / 7
*Age* (years)	70 (64−74)	68 (63−73)
*ECOG performance status* (0 / 1 / 2)	4 / 19 / 7	4 / 12 / 3
*Diagnosis*		
adenocarcinoma	15	9
squamous cell	11	8
large cell	0	1
undifferentiated	4	1
*Chemotherapy*		
carboplatin + vinorelbine	16	9
carboplatin + pemetrexed	12	8
Cisplatin	2	1
cisplatin + pemetrexed	0	1
*Medication*		
antihypertensive		
ACE inhibitor	3	5
β–blocker	3	2
Other	18	13
Antiplatelet	5	10
Diuretic	2	2
low molecular weight heparin	3	0
Statin	11	4
inhaled bronchodilator / corticosteroid	19	11
analgesic		
non-opioid	13	6
weak opioid	13	5
strong opioid	7	5
antidepressant (neuropathic pain)	1	1
anti-epileptic (neuropathic pain)	2	1
antidepressant	2	3
anxiolytic sedative	4	2
long-term oral corticosteroid	5	2
*Weight* (kg)	66.9 (59.9−76.0)	69.8 (61.1−80.1)
*Body mass index* (kg/m^2^)	27.0 (21.4−28.8)	25.1 (22.8−27.2)
*% weight loss*	0 (0−5.7)	0 (0−4.4)
*Nutritional intake*		
daily protein (g/day)	73 (64−88)	80 (65−100)
daily caloric intake (kcal/day)	1796 (1536−2179)	2077 (1708−2509)
*Body composition*		
whole body lean mass (kg)	47.5 (35.6−52.1)^a^	49.6 (36.2−53.1)^b^
*Met criteria for cachexia (n, %)*	(12, 40)	(12, 63)
low appendicular skeletal muscle index^c^	8	9
5% weight loss	8	5
body mass index <20kg/m^2^	3	1
*Quadriceps muscle strength* (kg)	16.4 (11.7−20.9)	20.5 (16.5−23.1)
*Mean daily physical activity level*		
step count	3146 (2040−3831)	3193 (1740−4644)
upright time (min)	232 (159−308)	194 (165−264)
up/down transitions	47 (35−60)	43 (33−58)
*Survival* (weeks)	27 [19-35]	23 [5-41]	

Data are median (IQR) or n,%.

^a^ based on 22 patients

^b^ based on 15 patients

^c^ males <7.26 kg/m^2^; females <5.45 kg/m^2^

Two of the 30 patients randomised to NMES rapidly deteriorated and died before receiving chemotherapy or the intervention. Of the remaining 28 patients, 10 did not complete three cycles of chemotherapy due to either clinical deterioration alone (n=2) or clinical deterioration and chemotherapy toxicities (n=8) which resulted in three further deaths. Three further patients withdrew due to NMES-related muscle discomfort (Figure 1). In comparison, of the 19 patients randomised to the control group, 6 did not complete three cycles of chemotherapy due to clinical deterioration which resulted in death (n=2) or early cessation of chemotherapy (n=4).

### Acceptability

Of the 28 patients who began NMES, seven reported initial muscle discomfort (CTC grade I) with three patients withdrawing from the study; the remaining four continued with NMES and the discomfort eased within a week.

Eighteen patients were eligible for the primary endpoint, i.e. 15 completing three cycles of chemotherapy together with the three who withdrew secondary to NMES-related muscle discomfort. Overall, nine (50% [90% CI, 29 to 71]) met the minimum adherence criterion of undertaking NMES three times weekly. Although two patients short from the required 20 patients in the original power calculation, for NMES to warrant further study would require ≥13 out of 18 to adhere (85% power).

The qualitative interviews revealed that adherence was enhanced by patients incorporating NMES sessions into a daily routine. Conversely, adherence was hindered by undesirable effects of chemotherapy, including hospitalisation, or when NMES sessions were perceived as not part of ‘normal’ life or interfered with social activity.

### Safety

There were no serious adverse events related to NMES. Six patients (21%) randomized to NMES were admitted to hospital during the study period. Precipitating factors were disease progression (n=2), infection, seizure (presenting feature of previously unknown brain metastases) and chemotherapy-related toxicity (one each of neutropenic sepsis, anaemia). In comparison, three patients (18%) in the control group were admitted to hospital because of cancer-related disseminated intravascular coagulation, infection or neutropenic sepsis.

### Physical performance

Fifteen patients in the NMES group and 13 patients in the control group completing three cycles of chemotherapy were available for analysis of the secondary outcome measures (Table 2). The proportion of patients in either group with stable disease (47% vs. 54%) or a partial response (33% vs. 38%) following chemotherapy were similar. A higher proportion of patients in the NMES group had progressive disease (20% vs. 8%) but this difference was not significant (p=0.76).

**Table 2:** Secondary outcomes expressed as median (IQR) (exploratory per-protocol analysis).

	*NMES (n=13)**^a^***			*Control (n=12)**^a^***			*p*
	*Baseline*	*Post-chemo*	*Change*	*Baseline*	*Post-chemo*	*Change*	
*Quadriceps muscle strength (kg)*	16.4 (11.7−20.9)	17 (13.1−19.3)	-0.1 (-1.9−2.7)	22 (18.4−23.1)	22 (19.2−25.4)	-2.1 (-3.4−2.7)	0.29
*Body composition*							
Thigh lean mass (kg)	6.1 (4.7−6.5)	6 (3.9−6.9)	0 (-0.4−0.6)	6.3 (4.2−6.4)	6.3 (4−6.6)	-0.2 (-0.3−0.2)	0.44
Whole body lean mass (kg)	48.9 (34.6,50.9)	45.4 (35.7,48.3)	-0.4 (-2.6−0.8)	49 (36.2−53.1)	51.2 (34−62.2)	-0.3 (-1.5−3.5)	0.31
*Mean daily physical activity level*							
Step count	3163 (2267−3855)	2766 (2053−4482)	-246 (-431−503)	3362 (2818−4644)	3332 (2636−4429)	51 (-1736−238)	1.00
Time upright (min)	268 (184−331)	208 (124−297)	-42 (-68−17)	190 (165−228)	160 (142−262)	-7 (-48−26)	0.38
Up/down transition	56 (45−73)	54 (42−65)	-5 (-13−5)	41 (33−61)	43 (37−59)	1 (-17−8)	0.66
*Fatigue (lower better)*							
General	13 (12−15)	13 (13−14)	-1 (-2−2)	11 (8−12)	13 (9−15)	1 (0−2)	0.14
Physical	13 (10−15)	13 (12−15)	0 (-1−2)	9 (5−14)	13 (11−15)	2 (0−7)	0.16
Reduced activity	16 (9−17)	12 (9−15)	0 (-1−1)	10 (5−16)	13 (10−14)	2 (-3−6)	0.26
Reduced motivation	10 (8−12)	8 (6−10)	-1 (-2−1)	10 (5−12)	10 (8−14)	2 (1−2)	0.08
Mental fatigue	9 (6−11)	8 (5−11)	0 (-4−2)	4 (4−7)	9 (5−12)	3 (0−6)	0.03
*Quality of life (higher better)*							
General health score	58 (42−67)	50 (50−67)	8 (-8−17)	67 (67−92)	67 (58−75)	-8 (-33−8)	0.22
Physical	73 (40−87)	67 (67−80)	0 (-7−13)	87 (73−93)	80 (60−87)	0 (-13−13)	0.47
Role	67 (50−83)	83 (66−100)	17 (0−17)	83 (67−100)	66 (66−83)	0 (-33−0)	0.06
Emotional	83 (75−92)	92 (67−100)	0 (0−8)	92 (92−100)	100 (75−100)	0 (-8−8)	0.71
Cognitive	83 (67−100)	83 (83−100)	0 (0−0)	100 (83−100)	83 (67−100)	0 (-17−0)	0.18
Social	83 (50−100)	100 (50−100)	0 (0−17)	100 (83−100)	100 (50−100)	0 (-33−0)	0.21

^a^ Only patients with both baseline and post-chemotherapy data included.

Compared to baseline, after three cycles of chemotherapy, there were no significant differences between the NMES and control groups in changes in quadriceps muscle peak strength, thigh lean mass or aspects of physical activity (Table 2). Although fatigue generally increased in both groups, there was a significant difference in the mental fatigue subscale (median change 0 vs. 3; p=0.03) favouring the NMES group, which would be considered clinically important (Table 2) [24].

Generally, quality of life deteriorated in the control group and remained unchanged in the NMES group. However, none of the changes were statistically significant between groups (Table 2).

## Discussion

The main finding of this phase II randomized controlled study is that NMES, based on our criteria, is not sufficiently acceptable alongside first-line palliative chemotherapy in patients with NSCLC to justify a phase III study in this setting. In addition, an exploratory per protocol analysis found no significant differences between the NMES and control groups with regard to quadriceps muscle strength, thigh lean mass or physical activity level. In the absence of the above, it is unlikely that the statistically significant difference found in the exploratory analysis of fatigue represents a specific effect of NMES.

Strengths of the study include an adequate sample size to explore the primary aim with sufficient power, the use of a programme of NMES shown to be effective in other settings [13], and a definition of adherence agreed *a priori*. A high uptake to the study by those approached (58%) increases the generalizability of the findings to this patient population. Our experience provides useful data with regards to recruitment and attrition rates for those planning similar supportive care studies alongside palliative chemotherapy. The secondary outcome assessment data and the embedded qualitative work can also be used to inform future trials.

The main weakness of the study is the reliance on patient reports of frequency and duration of use of NMES. Generally, patient reports of exercise undertaken tend to overestimate that actually done [32]. Thus, it could be that even those adhering to the programme overestimated their use of NMES and is one possible explanation why we found no overall benefit unlike others using a similar programme [13].

It could be argued that our adherence criteria were too rigid, e.g. a minimum of three NMES sessions per week, every week, and that our cut off to warrant further study was set too high, i.e. ≥80% of patients adhering. Certainly, three patients who failed to meet the minimum adherence criterion did so by missing only one session of NMES on one week. A more flexible overall minimum ‘dose’ criterion may be more appropriate for future studies. The high cut off reflects the level of adherence seen in our pilot study where daily use was recommended [15], and ensured that NMES would be performing similar or better in this regard compared to traditional exercise programmes [33].

Other potential limitations include the methods used to assess quadriceps muscle strength and bulk, i.e. hand-held dynamometry and DEXA scan [13]. Although more accurate assessments are available, e.g. fixed dynamometry [34] and magnetic resonance imaging [35], our selection was based on pragmatism, with a future multicenter study in mind. A lack of patient and assessor blinding could have introduced bias, although possible evidence for this is limited to non-significant differences in quality of life domains in the NMES group.

To our knowledge, this is the first study to examine the use of NMES alongside palliative chemotherapy and there are no directly comparable studies. In our randomized pilot study (n=16), when patients with advanced NSCLC who had completed chemotherapy were asked to use NMES 30 minutes daily for one month, median (range) reported adherence was higher at 80% (69–100). There was also more of a suggestion of global benefit from NMES with favourable changes in quadriceps strength, exercise endurance and physical activity, albeit statistically non-significant [15]. Possible explanations for the difference in findings include the populations studied and the treatment ‘dose’ of NMES. Although in the current study we explored the use of NMES earlier in the patients’ journey, paradoxically, this group had a poorer prognosis than those in the pilot study (i.e. median survival of 23 vs. 40 weeks) and thus, potentially less scope for benefit from NMES [15]. Patients received a lower ‘dose’ of NMES than in the pilot study. Although advised to use NMES ideally daily, in an attempt to ensure reasonable adherence over a longer period, we specified the minimum schedule associated with benefit, i.e. three treatments per week [13]. Our data suggest that unintentionally, many patients saw this as the target to aspire to and that the additional symptom and schedule burden of chemotherapy, along with periods of hospitalisation, contributed to a lower level of use.

Although a higher treatment ‘dose’ of NMES should theoretically be more beneficial, this is likely to be impractical given the challenges this group face with regard to morbidity related to their disease and chemotherapy. In this regard, NMES is unlikely to offer any distinct advantage over other traditional forms of exercise. Further, given we found little decline in our secondary outcome measures in those patients completing three cycles of treatment, the rationale for the use of NMES alongside chemotherapy, at least for this patient group receiving these treatment regimens, could be questioned.

Nonetheless, the need remains for adequately powered studies to examine if NMES can provide meaningful benefit to patients with NSCLC in other settings, either given alone, or as part of a combined approach, e.g. with nutritional support, anti-inflammatory drug or more specific anti-cachexia treatment [36]. Based on our findings, we would suggest patients are recruited after completing chemotherapy and that daily treatment is advised, rather than a minimum.

In conclusion, based on our criteria, NMES is not an acceptable exercise intervention alongside first-line palliative chemotherapy in patients with NSCLC. Poor adherence and high rates of attrition contribute towards a lack of overall benefit in this setting. The need remains to identify if NMES is of benefit in patients with cancer in other settings.

## Supporting Information

Checklist S1 **CONSORT Checklist.**
(DOCX)

Protocol S1 **Trial protocol.**
(PDF)
