# Supplementary material for: Neuromuscular Electrical Stimulation of the Quadriceps in Patients with Non-Small Cell Lung Cancer Receiving Palliative Chemotherapy: A Randomized Phase II Study
Source: PLoS One. 2013 Dec 30;8(12):e86059. doi: 10.1371/journal.pone.0086059 (PMC3875585; doi:10.1371/journal.pone.0086059)
Supplement: Protocol S1 — Trial protocol. (PDF) [file pone.0086059.s002.pdf]

Protocol S1.

NMES

NMES for patients with NSCLC receiving palliative chemotherapy

Is neuromuscular electrical stimulation an acceptable and feasible supportive therapy for patients with non-small cell lung cancer receiving palliative chemotherapy?

Clinical Trial Protocol

Version: 4.0

Date: 23rd January 2011

ISRCTN No.: 42944026

Funder: National Cancer Research Institute

Funder No: LCSuPaC 35

Name of Sponsor: Nottingham University

Developed on behalf of the Cachexia subgroup of the NCRI Palliative Care Clinical Studies Group

General Information

This protocol describes the NMES clinical trial, and provides information about the procedures for entering patients into the trial. The protocol should not be used as a guide, or as an aide-memoire for the treatment of other patients. Every care has been taken in drafting this protocol; however, corrections or amendments may be necessary. These will be circulated to the known investigators in the trial, but centres entering patients for the first time are advised to contact the Wales Cancer Trials Unit in Cardiff to confirm that they have the most up-to-date version of the protocol in their possession. Problems relating to the trial should be referred, in the first instance, to the Wales Cancer Trials Unit.

Compliance

This trial will adhere to the conditions and principles which apply to all clinical trials as outlined in the ICH Harmonised Tripartite Guideline for Good Clinical Practice (CPMP/ICH/135/95). It will be conducted in compliance with the protocol; the Research Governance Framework for Health and Social Care (Welsh Assembly Government November 2001 and Department of Health 2nd July 2005); the Data Protection Act 1998 and other regulatory requirements as appropriate.

Funding

The NMES trial is being funded by the National Cancer Research Institute Supportive and Palliative Care Lung Cancer Initiative, on behalf of the National Cancer Research Institute and thus is part of the NCRN portfolio of clinical trials.

RANDOMISATION Telephone Number:

029 2064 5500

Open 9am to 5pm (Monday to Friday)

Serious Adverse Event (SAE) Fax Number:

029 2064 4488

Trial Coordination

The NMES trial is being coordinated by the Wales Cancer Trials Unit (WCTU), a Cardiff University hosted, National Cancer Research Institute (NCRI) accredited, and United Kingdom Clinical Research Collaboration (UKCRC) registered trials unit.

This protocol has been developed by the NMES Trial Management Group (TMG).

Wales Cancer Trials Unit	Tel: +44 (0) 29 2068 7500
6th Floor	Fax: +44 (0) 29 2068 7501
Neuadd Meirionnydd	
University Hospital of Wales	Email: NMES@cardiff.ac.uk
Heath Park	Website: www.wctu.org.uk
Cardiff	
CF14 4YS	

WCTU NMES trial staff

**Trial Manager:**	**Mrs Sarah Bridges**	**Tel: +44 (0) 29 2068 7463**<br>**Email: BridgesSE@wctu.cf.ac.uk**
Scientific Director:	Mr. Gareth Griffiths	Tel: +44 (0) 29 2968 7456<br>Email: GriffithsG@wctu.cf.ac.uk
Scientific Lead:	Dr Anthony Byrne	Tel: +44 (0) 2968 7476<br>Email: Anthony.byrne2@wales.nhs.uk
Senior Statistician:	Mr Chris Hurt	Tel: +44 (0) 29 2068 7471<br>Email: HurtCN@wctu.cf.ac.uk
Senior Research Fellow:	Dr Annmarie Nelson	Tel: +44 (0) 29 2068 7473<br>Email: annmarie.nelson@wctu.cf.ac.uk
Safety Desk		Tel: +44 (0) 29 2068 7469<br>Email: WCTU-safety@cardiff.ac.uk

For all queries please contact the NMES Trial Manager. Any clinical queries will be directed through the Trial Manager to either the Chief Investigator or one of the clinical Co-Investigators.

Chief Investigator

Dr Andrew Wilcock
Clinical Reader in Palliative Medicine and Medical Oncology
Hayward House Macmillan Specialist Palliative Cancer Care Unit
Nottingham University Hospitals NHS Trust
City Hospital Campus
Hucknall Road
Nottingham
NG5 1PB

Co-Investigators

Mr Matthew Maddocks
Research Physiotherapist
Clinical Sciences Building
Nottingham University Hospitals NHS Trust
City Hospital Campus
Nottingham
NG5 1PB

Mr Gareth Griffiths
Scientific Director
Wales Cancer Trials Unit
6th Floor, Neuadd Meirionnydd
University Hospital of Wales
Cardiff
CF14 4YS

Dr Anthony Byrne
Scientific Lead
Wales Cancer Trials Unit
6th Floor, Neuadd Meirionnydd
University Hospital of Wales
Cardiff
CF14 4YS

Mrs Vanessa Halliday
Teacher Practitioner Dietetics
Room 49F, North Lab
Nutritional Sciences
Sutton Bonington Campus
University of Nottingham
LE12 5RD

Advisors

Dr Annmarie Nelson
Marie Curie Palliative Care Senior Research Fellow
Wales Cancer Trials Unit
6th Floor, Neuadd Meirionnydd
University Hospital of Wales
Cardiff
CF14 4YS

Dr Nicola Adams
Lecturer in Physiotherapy
University of Nottingham
Division of Physiotherapy Education
City Hospital Campus
Nottingham
NG5 1PB

Mr Andy Brown
Head of Medical Physics
Dept of Medical Physics and Clinical Engineering
Nottingham University Hospitals NHS Trust
City Campus
Hucknall Rd
Nottingham
NG5 1PB

Dr Des Green
Consultant Radiologist
Nottingham University Hospitals NHS Trust
City Campus
Hucknall Rd
Nottingham
NG5 1PB

Consumer representative
Mrs Joyce Calum
Palliative Care Clinical Studies Group
NCRN Coordinating Centre
University of Leeds
24 Hyde Terrace
Leeds
LS2 9LN

Table of contents

Contents

Trial Coordination ... 2
Table of contents ... 4
Abbreviations and glossary ... 5
1.0 Trial schema ... 7
2.0 Trial synopsis ... 8
2.1 Lay summary ... 11
3.0 Background, rationale and objectives ... 12
4.0 Study design ... 13
5.0 Participating centre selection ... 14
6.0 Participant eligibility ... 15
6.1 Inclusion criteria ... 15
6.2 Exclusion criteria ... 15
6.3 Participant recruitment ... 15
6.4 Informed consent ... 15
7.0 Randomisation ... 16
8.0 Treatments ... 17
8.1 Control arm ... 17
8.2 NMES arm ... 17
9.0 Trial assessments ... 18
9.1 Baseline assessments ... 18
9.2 Ongoing assessments during NMES ... 18
9.3 End of treatment assessments (9 weeks from start of chemotherapy) ... 18
9.4 End of treatment assessments for patients proceeding to cycle 4 (12 weeks from start of chemotherapy) ... 18
9.5 Follow up assessments (17 / 20 weeks from start of chemotherapy) ... 18
9.6 Schedule of trial ... 20
10.0 Safety reporting ... 21
10.1 Pregnancy reporting whilst participating in the NMES trial ... 22
10.2 Exceptions ... 22
10.3 Centre responsibilities ... 23
10.4 The Wales Cancer Trials Unit responsibilities ... 23
10.5 Flowchart for Serious Adverse Event reporting ... 24
11.0 Trial conduct and monitoring ... 25
11.1 Monitoring and quality assurance ... 25
11.2 Central monitoring ... 25
11.4 Trial closure ... 26
12.0 Statistical considerations ... 27
12.1 Randomisation ... 27
12.2 Outcome measures ... 27
12.3 Sample size calculation ... 27
12.4 Interim analyses and the role of the Independent Data Monitoring Committee ... 28
12.5 Statistical analyses ... 28
12.6 Subgroup analyses ... 28
13.0 Sub-studies ... 29
Qualitative interview study. ... 29
14.0 Publication policy ... 31
15.0 Informed consent, ethical and regulatory considerations ... 32
15.1 Ethical and other issues ... 32
15.3 Sponsorship ... 33
15.4 Indemnity ... 33
15.5 Data protection ... 34
15.6 Finance ... 34
16.0 Trial committees and trial management arrangements ... 35
17.0 References ... 36
APPENDIX 1: Interview schedule ... 37

Abbreviations and glossary

**AE**	Adverse Event
**CI**	Chief Investigator
**COPD**	Coronary Obstructive Pulmonary Disease
**CRF**	Case report form
**CTCAE**	Common Terminology Criteria for Adverse Events
**DEXA**	Dual Energy X-ray Absorptiometry
**ECOG**	Eastern Cooperative Oncology Group
**GCP**	Good Clinical Practice
**GP**	General practitioner
**ICH**	International Conference on Harmonisation
**ICH-GCP**	International Conference on Harmonisation – Good Clinical Practice
**IDMC**	Independent Data Monitoring Committee
**ISF**	Investigator Site File
**ISRCTN**	International Standard Randomised Controlled Trial Number
**LREC**	Local Research Ethics Committee
**MHRA**	Medicines and Healthcare products Regulatory Agency
**NCRI**	National Cancer Research Institute
**NCRN**	National Cancer Research Network
**NHS**	National Health Service
**NMES**	Neuromuscular electrical stimulation
**NSCLC**	Non-small cell lung cancer
**Patient**	A patient under care who may be eligible for the trial but has not yet consented to participate in any trial related activities.
**Participant**	An individual who has given written informed consent and is participating in trial related activities
**PI**	Principal Investigator
**PIS**	Participant Information Sheet
**R&D**	Research and Development
**REC**	Research Ethics Committee
**SAE**	Serious Adverse Event
**SOP**	Standard Operating Procedure
**SSA**	Site-specific assessment

**TMF**	Trial Master File
**TMG**	Trial Management Group
**TSC**	Trial Steering Committee
**TSF**	Trial Site File
**UKCRC**	United Kingdom Clinical Research Collaboration
**WCTU**	Wales Cancer Trials Unit

1.0 Trial schema

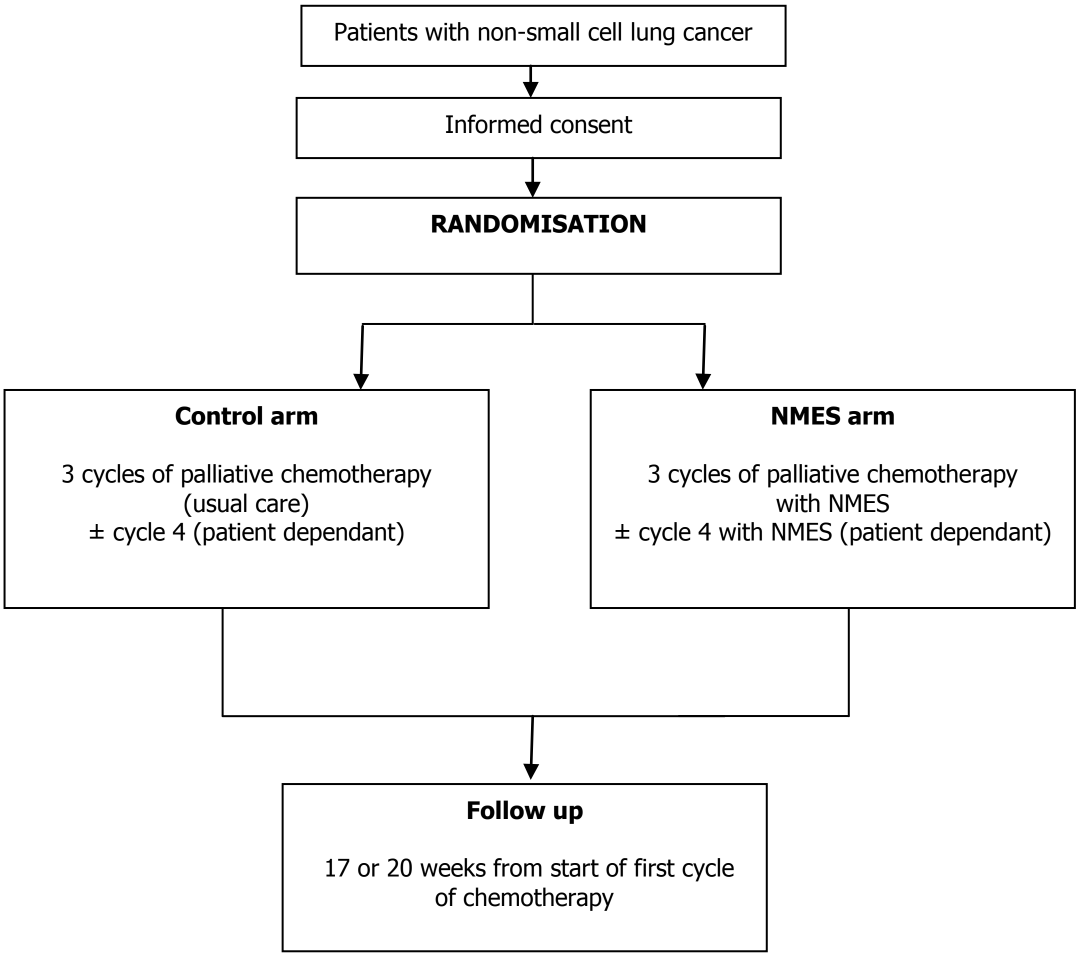

Primary outcome measure:

- *Adherence to NMES*: self-report daily diary of length of time NMES used and internal logger recording total and individual periods of use.

Secondary outcome measures:

- *Safety of NMES:* toxicities will be assessed according to the NCI Common Terminology Criteria for Adverse Events (NCI CTCAE v3.0). Serious adverse events will be monitored in "real-time".
- *Quadriceps muscle strength*: peak torque (kg) measured using a portable Manual Muscle Tester dynamometer (Lafayette Instruments, USA).
- *Body composition*: lean tissue mass of the body and thighs (kg) assessed by low-dose dual energy x-ray absorptiometry (DEXA).
- *Physical activity level*: mean daily step count assessed by a small, lightweight activPAL™ accelerometer worn on the thigh for one week.
- *Nutritional intake*: mean daily energy (kJ/d) and protein (g/d) intake estimated from a 3-day food and drink diary.
- *Fatigue*: Multidimensional Fatigue Inventory.
- *Quality of life*: the EORTC-C30 and the lung cancer specific module LC-13.
- *Experience of NMES* - semi-structured interview exploring patient experiences of completing NMES during chemotherapy.

2.0 Trial synopsis

**Study title:**	Is neuromuscular electrical stimulation an acceptable and feasible supportive therapy for patients with non-small cell lung cancer receiving palliative chemotherapy?				
**Study acronym:**	NMES				
**Short title:**	NMES for patients with NSCLC receiving palliative chemotherapy				
**ISRCTN No:**	42944026				
**Funder:**	National Cancer Research Institute	Funder's No:		LCSuPaC 35	
**Chief Investigator:**	Dr Andrew Wilcock				
**Sponsor:**	Nottingham University				
**Study period:**	2 years	Phase:	II	No. of arms:	2
**No. of participants:**	47				

Objectives

Primary: Is NMES an acceptable and feasible supportive therapy for patients with non-small cell lung cancer undergoing at least 3 cycles of palliative chemotherapy?

Secondary:
i) Is NMES a safe intervention to offer patients undergoing palliative chemotherapy?
ii) To what extent does 3 cycles of palliative chemotherapy impact on leg muscle strength, body composition and physical activity levels in patients with NSCLC and can the use of NMES influence these changes?
iii) What is the rate of recovery or decline in these parameters following cessation of 3–4 cycles of chemotherapy and can the use of NMES influence this?
iv) What are patients' attitudes about the use of NMES during chemotherapy?

Main inclusion criteria:

- Equal or greater than 16 years of age
- Histological diagnosis of non small cell lung cancer
- ECOG performance status of 0, 1 or 2
- Scheduled to receive 3–4 cycles of first line palliative chemotherapy
- Able to use the NMES device
- Able to provide written informed consent to participate in trial.

Main exclusion criteria:

- Implanted cardiac pacemaker
- Epilepsy
- Spinal cord pathology
- Pregnancy

Treatments:

Control arm

3–4 cycles of first line palliative chemotherapy administered as part of usual care.

NMES arm

3–4 cycles of first line palliative chemotherapy with carboplatin/vinorelbine administered as part of usual care plus NMES for 30min daily at home to the anterior thighs throughout chemotherapy.

Trial assessments:

Baseline assessments:
Quadriceps muscle strength
Body composition
Physical activity level
Nutritional intake
Fatigue
Quality of life

Ongoing assessments during NMES programme:
Adherence to NMES
Safety of NMES

9 weeks from start of chemotherapy assessments:
Quadriceps muscle strength
Body composition
Physical activity level
Nutritional intake
Fatigue
Quality of life
Experience of NMES

12 weeks from start of chemotherapy assessments (if cycle 4 received):
Experience of NMES

17/20 week from start of chemotherapy assessments (3/4 cycles received)
Quadriceps muscle strength
Body composition
Physical activity level
Nutritional intake
Fatigue
Quality of life

Endpoints:

Primary outcome measure:

- *Adherence to NMES*: Proportion of patients completing a pre-determined level of compliance to the recommended NMES programme measured using a self-report diary.

Secondary outcome measures:

- Safety of NMES*:* toxicities will be assessed according to the NCI Common Terminology Criteria for Adverse Events (NCI CTCAE v3.0). Serious adverse events will be monitored in "real-time".
- Quadriceps muscle strength: peak torque (Nm) measured using a portable Manual Muscle Tester dynamometer (Lafayette Instruments, USA).
- Body composition: lean tissue mass of the body and thighs (kg) assessed by low-dose dual energy x-ray absorptiometry (DEXA).

- Physical activity level: mean daily step count assessed by an activPAL™ monitor over 1 week.
- Nutritional intake: mean daily energy (kJ/d) and protein (g/d) intake estimated from a 3-day (2 weekdays, 1 weekend day) food and drink diary.
- Fatigue: Multidimensional Fatigue Inventory.
- Quality of life: the EORTC-C30 and LC-13.
- Experience of NMES - qualitative sub-study. A semi-structured interview exploring patients experiences of completing NMES during chemotherapy.

2.1 Lay summary

People with lung cancer receive chemotherapy to improve symptoms and survival. However, chemotherapy can make leg muscles weak. The reason is unclear, but may relate to reduced activity due to fatigue. Maintaining leg muscle strength is important as this helps determine how active and independent a person is.

Exercise helps maintain leg muscle strength, but even simple forms, e.g. walking, can be difficult when fatigued. An alternative form of exercise is the use of neuromuscular electrical stimulation (NMES). A small battery operated stimulator with two pads placed over each thigh produces a controlled, comfortable, slow contraction and relaxation of the underlying muscles while the person is seated. Our group recently found that NMES 30min daily for one month improved leg muscle strength and physical activity levels in people with lung cancer not on chemotherapy.

NMES looks a promising therapy. However, to determine if NMES is worth pursuing, we wish to examine if it is acceptable to a larger group of people. Given that chemotherapy weakens leg muscles, it is also appropriate that we examine its use in this setting. We would also like to explore further why chemotherapy causes leg muscle weakness.

47 people due to receive chemotherapy will be required for the study. One third will be randomised to receive usual care and two thirds to receive usual care plus NMES. The NMES group will be asked to use the treatment on both legs daily throughout their 3–4 cycles of chemotherapy. Acceptability of NMES will be assessed by diary records of how much the stimulator is used. The potential benefit of NMES will be explored by assessing leg muscle strength, muscle size and physical activity levels, and asking people about their experiences. We will also explore why leg muscles become weaker during chemotherapy by assessing fatigue and nutritional factors and documenting the response of the cancer to chemotherapy. The intervention will be monitored for safety in real time.

3.0 Background, rationale and objectives

Palliative chemotherapy is offered to patients with non-small cell lung cancer (NSCLC) to improve symptoms, quality of life and survival.[1] However, not all patients benefit and chemotherapy, contrary to the above aim, can lead to a deterioration in leg muscle strength and physical activity levels.[2-4] The reason is unclear, but may relate to fatigue causing reduced activity and muscle deconditioning. In order to help patients remain as independent as possible, new approaches are required to help minimise this negative effect of chemotherapy.

One option is exercise.[5] However, in patients with incurable cancer, traditional forms of exercise are limited by low rates of uptake and adherence.[6,7] Alternative forms of exercise are needed which require less motivation and change in lifestyle. One approach is neuromuscular electrical stimulation (NMES). A small battery operated stimulator with two skin electrodes placed over each thigh produces a controlled, comfortable, slow contraction and relaxation of the underlying muscles while the person is seated. This has improved muscle strength and exercise capacity in patients with COPD and heart failure.[8,9]

The Nottingham group has just completed a pilot study of NMES in patients with incurable NSCLC not receiving chemotherapy (n=16). Patients were randomised to undertake NMES to each thigh for 30min daily for one month or to a control group which received usual care. NMES appeared acceptable and was used for a median (range) of 80% (69–100) of the total recommended time. Change from baseline in leg muscle strength, physical activity (daily step count) and exercise endurance (endurance shuttle walk test) revealed a trend towards benefit from NMES.

Thus, NMES appears worthy of further examination as a supportive therapy for cancer-related cachexia. However, because chemotherapy also weakens leg muscles, it is appropriate that we explore the potential benefit of NMES in this setting before embarking on a phase III trial. We will also explore why leg muscles become weaker during chemotherapy by assessing fatigue and nutritional factors and documenting the response of the cancer to chemotherapy.

Overall aim
To provide data which will help determine the feasibility and justification for a phase III trial of NMES.

Primary objective
Is NMES an acceptable and feasible supportive therapy for patients with non-small cell lung cancer undergoing at least 3 cycles of palliative chemotherapy?

Secondary objectives
i) Is NMES a safe intervention to offer patients undergoing palliative chemotherapy?

ii) To what extent does 3-4 cycles of palliative chemotherapy impact on leg muscle strength, body composition and physical activity levels in patients with NSCLC and can the use of NMES influence these changes?

iii) What is the rate of recovery or decline in these parameters following cessation of 3–4 cycles of chemotherapy and can the use of NMES influence this?

iv) What are patients' attitudes to the use of NMES during chemotherapy?

4.0 Study design

An open randomised phase II study in patients with non-small cell lung cancer. 47 patients will be randomised to receive either palliative chemotherapy or palliative chemotherapy plus the NMES programme. Patients will be randomised with a 2:1 allocation ratio in favour of the NMEs intervention arm.

A Flemings one-stage design is applied to the experimental arm, with the proportion of patients completing NMES for 30 minutes, three times a week as the primary endpoint.

A control arm will be used to see what the change in activity is (muscle strength, body composition, physical activity, safety) in a similar population of patients having usual practice of palliative chemotherapy.

5.0 Participating centre selection

This study will be carried out at the City Hospital Campus of Nottingham University NHS Trust.

The following documentation must be received by the WCTU in order for this centre to begin recruitment:

- Confirmation of local research ethics committee (LREC) approval (Site-specific assessment)
- Confirmation of local R&D approval
- Documentation of delegated responsibilities to PI at site
- Current Curriculum Vitae of the PI
- A copy of the most recent version of the Participant Information Sheet and Consent Form on host care organisation headed paper
- Completed Investigator Statement (signed and dated by the PI)
- Completed Delegation Log (signature list and delegation of responsibilities)
- Full contact details for all host care organisation personnel, indicating preferred contact
- Written confirmation that the PI will ensure that participants are competent to administer the trial treatment

Once all the documentation has been received at the WCTU, confirmation of centre approval will be sent by the WCTU to the centre PI.

All documentation must be stored in the Investigator Site File (ISF) at the site and in the Trial Site File (TSF) at the WCTU. The WCTU must be notified of any changes to the trial personnel and their responsibilities during the running of the trial and the respective trial files must contain this up-to-date information.

6.0 Participant eligibility

A total of 47 participants are required for the NMES trial.

Any queries about whether a patient is eligible to enter the trial should be discussed with the WCTU before randomisation. Concerns will then be raised with the Chief Investigator (CI) or one of the clinical Co-Investigators in the CI's absence.

Patients are eligible for the trial if all the inclusion criteria (Section 6.1) are met and none of the exclusion criteria (Section 6.2) apply:

6.1 Inclusion criteria

Patients meeting all of the following criteria may be included in the trial:

- Aged 16 years or older
- ECOG Performance Status score of 0, 1 or 2
- Is scheduled to receive 3–4 cycles of first line palliative chemotherapy
- Able to use NMES device
- Life expectancy > 3 months
- Has provided written informed consent.

The researcher must confirm the eligibility of a patient in the patient's medical notes prior to randomisation.

6.2 Exclusion criteria

If any of the following criteria apply, patients cannot be included in the trial:

- Patient has an implanted cardiac pacemaker
- Patient has epilepsy
- Patient has spinal cord pathology
- Is pregnant or breastfeeding

6.3 Participant recruitment

Eligible patients will be identified through MDT meetings and screening of patients medical notes by members of the research team who will be the clinicians normally providing their routine clinical care.

Patients' usual clinicians will be asked to offer a patient information sheet during a routine consultation. Acceptance of the patient information sheet is entirely voluntary. If patients take an information sheet, a member of the research team will subsequently contact them via telephone.

6.4 Informed consent

The patient's written informed consent must be obtained using the NMES trial Consent Form, which follows the Participant Information Sheet. The patient should be given a minimum of 24 hours after the initial invitation to participate before being asked to sign the Consent Form. Please note, only when written informed consent has been obtained from the patient can they be considered a trial participant.

7.0 Randomisation

Randomisation will be performed centrally by the WCTU. Randomisation can only be performed once the participant has signed the Consent Form. The randomisation form should be completed and the WCTU contacted on the following telephone number:

WCTU Randomisation line:
029 2064 5500
(Open Monday – Friday, 9am – 5pm)

N.B. This telephone number is strictly for randomisation and should not be used for general queries.

Participants will be randomised to a trial arm using a 2:1 allocation ratio using balanced blocks. At randomisation, the participant will be given a unique participant trial number. These details should be recorded on the NMES participant randomisation form and the top copy returned to the WCTU within four weeks.

After randomisation, the WCTU will fax confirmation to the Research Physiotherapist at the participating centre. Case Report Forms (CRFs) will contain the participant trial number, initials and date of birth. CRFs will also be sent to the Clinician, Data Manager or Research Nurse nominated as responsible for the participant. The participant's General Practitioner (GP) will be informed of the participant's randomisation, if the participant gives consent to do so.

It may be possible for participants to be recruited into other clinical trials, but this should be discussed with the WCTU.

8.0 Treatments

Participants will be allocated to receive one of the following treatment regimen(s):

8.1 Control arm

3–4 cycles of first line palliative chemotherapy administered as part of usual care by Nottingham University Hospital NHS Trust staff

8.2 NMES arm

3–4 cycles of first line palliative chemotherapy administered as part of usual care by Nottingham University Hospital NHS Trust staff and NMES programme.

NMES programme

NMES for 30min daily at home to the anterior thighs throughout chemotherapy. Because patients are generally anxious about starting chemotherapy we will defer initiating NMES until one week after commencing cycle one.

A physiotherapist will supervise the first session of NMES either at hospital or in the patient's home depending on patient preference. This will be supplemented by written instructions, weekly phone calls and home visits if required. When conducting home visits the physiotherapist will follow the WCTU home safety procedure SOP and a buddy system SOP.

Stimulation will be delivered using a MicroStim Exercise Stimulator MS2v2 (Odstock Medical Ltd, Wiltshire, UK) and self-adhesive electrodes placed over the body of the quadriceps. This is a CE marked (CE 02104) Class IIa Medical Device and the application is in accordance with the CE marking reference for the device.

The proportion of the treatment duration which is active, i.e. the stimulation phase of the duty cycle, will increase on a weekly basis from 11% to 18% to 25%, remaining constant thereafter.

The intensity or amplitude (device output 0–120mA, tested across 1000Ω) will initially be set to elicit a visible and comfortable muscle contraction.

Thereafter, patients will be encouraged to increase the amplitude as tolerated during the monitoring phone calls.

9.0 Trial assessments

9.1 Baseline assessments

- Quadriceps muscle strength: peak torque (Kg) measured using a portable Manual Muscle Tester dynamometer (Lafayette Instruments, USA).
- Toxicities
- Body composition: lean tissue mass of the body and thighs (kg) assessed by low-dose dual energy x-ray absorptiometry (DEXA) maximum dose per scan 37.4μSv.
  The radiation dose for this procedure has been reviewed by a Medical Physics Expert and a Consultant Radiologist (page 3) and is considered to present 'trivial' and 'very low' risk.
- Physical activity level: mean daily step count assessed by an ActivPAL™ monitor over 1 week
- Nutritional intake: mean daily energy (kJ/d) and protein (g/d) intake estimated from a 3-day food and drink diary (2 weekdays, 1 weekend day)
- Fatigue: Multidimensional Fatigue Inventory
- Quality of life: the EORTC-C30 and LC-13.

9.2 Ongoing assessments during NMES

- Adherence to NMES: self-report daily diary of length of time NMES used and, if possible, an internal logger recording individual and total number of sessions used.
- Safety of NMES: toxicities and real-time adverse event reporting using CTCAE v3.0 criteria.

9.3 End of treatment assessments (9 weeks from start of chemotherapy)

- Quadriceps muscle strength
- Toxicities
- Body composition
- Physical activity level
- Nutritional intake
- Fatigue
- Quality of life
- *Response to chemotherapy:* overall objective clinical response categorised using Response Evaluation Criteria In Solid Tumours, documented from patients' medical reports.
- Experience of NMES - <u>only in those that agree to participate in the qualitative sub-study and are not scheduled to proceed to 4 cycles of chemotherapy</u>. A semi-structured interview exploring patients experiences of completing NMES during chemotherapy.

9.4 End of treatment assessments for patients proceeding to cycle 4 (12 weeks from start of chemotherapy)

- *Response to chemotherapy:* overall objective clinical response categorised using Response Evaluation Criteria In Solid Tumours, documented from patients' medical reports.
- Experience of NMES - <u>only in those that agree to participate in the qualitative sub-study and receive 4 cycles of chemotherapy</u>. A semi-structured interview exploring patients experiences of completing NMES during chemotherapy.

9.5 Follow up assessments (17 / 20 weeks from start of chemotherapy)

- Quadriceps muscle strength
- Toxicities
- Body composition
- Physical activity level
- Nutritional intake
- Fatigue
- Quality of life.

A detailed assessment schedule is given in section 9.6 overleaf.

9.6 Schedule of trial

**Procedure / Assessment**	**Trial time-point**													
	Week -1	Week 1	Week 2	Week 3	Week 4	Week 5	Week 6	Week 7	Week 8	Week 9	Week 10	Week 11	Week 12	Week 17/20
Chemotherapy		X			X			X			X			
Response documented										X			X	
Toxicities	X		X	X	X	X	X	X		X			X	X
Assessments														
Leg muscle strength	X									X				X
Physical activity level	X									X				X
Body composition	X									X				X
Nutritional status	X									X				X
Fatigue	X									X				X
Quality of life	X									X				X
NMES														
Daily application			X	X	X	X	X	X	X	X	X	X	X	
Supervision (home visit)			X						X					
Supervision (telephone call)				X	X	X	X	X			X	X	X	
Adherence assessed										X			X	
Interview (subgroup)										X			X	

N.B. Serious Adverse Events (SAE) will be collected in real time via a designated SAE fax number.

Notes: the timing of assessments will be standardised to treatment cycles and thus will be sufficiently flexible to cope with delays during chemotherapy, e.g. due to neutropenia. The interview is completed either at week 9 or week 12 depending on the patient receiving 3 or 4 cycles of chemotherapy respectively.

10.0 Safety reporting

The following definitions are in accordance with ICH-GCP.

Adverse Event (AE): Any untoward medical occurrence in a clinical trial participant which does not necessarily have a causal relationship with their treatment. An AE can therefore be any unfavourable and unintended sign (including abnormal laboratory finding), symptom, or disease.

An adverse event that causes, or has the potential to cause, unexpected or unwanted effects involving the safety of patients, users or other persons can include:

- A patient, carer or professional is injured as a result of a medical device failure or misuse
- A patient's treatment is interrupted or compromised by medical device failure
- A patient's health deteriorates due to medical device failure

An adverse event in medical devices may arise due to:

- Shortcomings in the manufacture or design of the device itself
- Inadequate instructions for use
- Inadequate servicing or maintenance
- Locally initiated modifications or adjustments
- Inappropriate user practices (which could arise from inadequate training)
- The environment in which the device is used or stored

Adverse events should be reported to the WCTU to identify the rate at which they occur and the severity of outcome.

Adverse events may include any noxious and unintended response in a clinical trial participant to whom an investigational medicinal device has been administered, which is related to any intensity level administered.

An adverse event with a medical device means that a causal relationship between a medical device and is at least a reasonable possibility, i.e. the relationship cannot be ruled out.

The expected adverse reactions for Microstim 2 (v2) are tabulated below:

• Post-NMES muscle soreness
• Erythema following NMES
• Rash on skin in reaction to electrodes

Serious Adverse Event (SAE): Any adverse event that:

- Results in death
- Is life-threatening*
- Required hospitalisation or prolongation of existing hospitalisation**
- Results in persistent or significant disability or incapacity
- Consists of a congenital anomaly or birth defect
- Other medically important condition ***

* Note: The term "life-threatening" in the definition of serious refers to an event in which the patient was at risk of death at the time of the event; it does not refer to an event which hypothetically might have caused death if it were more severe.

** Note: Hospitalisation is defined as an inpatient admission, regardless of the length of stay, even if the hospitalisation is a precautionary measure, for continued observation. Pre-planned hospitalisation e.g. for pre-existing conditions which have not worsened or elective procedures does not constitute an adverse event.

*** Note: other events that may not result in death, are not life-threatening, or do not require hospitalisation may be considered as a serious adverse event when, based upon appropriate medical judgement, the event may jeopardise the participant and may require medical or surgical intervention to prevent one of the outcomes listed above.

Adverse events (AE) and AIs should be graded using the NCI Common Terminology Criteria for Adverse Events (CTCAE) Version 3.0. The toxicity grades should be recorded on the CRF.

A SAE form is not considered as complete unless the following details are provided:

- Full participant trial number
- An adverse event/reaction
- A completed assessment of the seriousness, causality and expectedness as performed by the Principal Investigator or another appropriately qualified clinician registered on the delegation log

If any of these details are missing, you will be contacted and the information must be provided as soon as it becomes available.

10.1 Pregnancy reporting whilst participating in the NMES trial

Pregnancy, occurring whilst participating in the NMES trial, although not considered an SAE, must be notified to the WCTU within the same timelines as an SAE. In the event of a pregnancy in a trial participant, the WCTU must be contacted immediately to request a Pregnancy Report Form. The Pregnancy Report Form should be completed and returned to the WCTU to capture all the relevant information required for the expedited reporting of these events. The outcome of a pregnancy should be followed up carefully and any abnormal outcome of the mother or the foetus should be reported.

10.2 Exceptions

For the purposes of this trial the following SAEs do not require immediate reporting.

- Death or disability due to disease progression
- Hospitalisation for chemotoxicities, e.g neutropenia, infection, nausea
- Toxicities related to palliative radiotherapy

Instead, these should be completed on the relevant CRF page and forwarded to the WCTU in the normal timeframes for CRFs (i.e. within two weeks of completion).

10.3 Centre responsibilities

All SAEs must be reported immediately by the PI at the participating centre to the WCTU and the Chief Investigator unless the SAE/SAR is specified as not requiring immediate reporting (see above). All other AEs should be reported on the CRF as usual. The PI should assess the SAE to determine the likely causality with the trial treatment (graded as definitely, probably, possibly, unlikely or not related) and the expectedness (unexpected or expected). A completed SAE form for all events requiring immediate reporting should be faxed to the WCTU and University of Nottingham within 24 hours of knowledge of the event. A separate form must be used to report each event.

SAE Fax Number:

029 2064 4488

It is also required that sites respond to and clarify any queries raised on any reported SAEs and report any additional information as and when it becomes available through to the resolution of the event.

Serious adverse events should continue to be reported until 30 days after the last patient receives their last dose of the investigational medicinal product. Serious adverse reactions must continue to be reported until the end of follow up.

10.4 The Wales Cancer Trials Unit responsibilities

Following the initial report all SAEs should be followed up to resolution wherever possible and further information may be requested by the WCTU. The participant will be identified only by trial number, date of birth and initials. The participant's name should not be used on any correspondence.

WCTU has been delegated responsibility for reporting AEs and SAEs to the regulatory authorities (and relevant ethics committees) as follows:

- A list of all AEs (expected and unexpected) will be reported annually to the Main REC and the trial sponsor.
- The WCTU will report a list of all SARs (expected and unexpected) and any other safety recommendations to all PIs every six months throughout the course of the trial. This frequency may be reviewed and amended as necessary.

Once an SAE or AE is received at the WCTU, it will be evaluated by staff at the WCTU and the Chief Investigator (or their delegate) for seriousness, expectedness and causality.

The causality and expectedness assessment given by the PI cannot be overruled by the CI (or their delegate) and in the case of disagreement, both opinions will be provided with the report. SAEs will be handled at the highest level of event categorisation. This ensures that all SAEs will be reported correctly and included into any Annual / Investigator Safety Reports.

10.5 Flowchart for Serious Adverse Event reporting

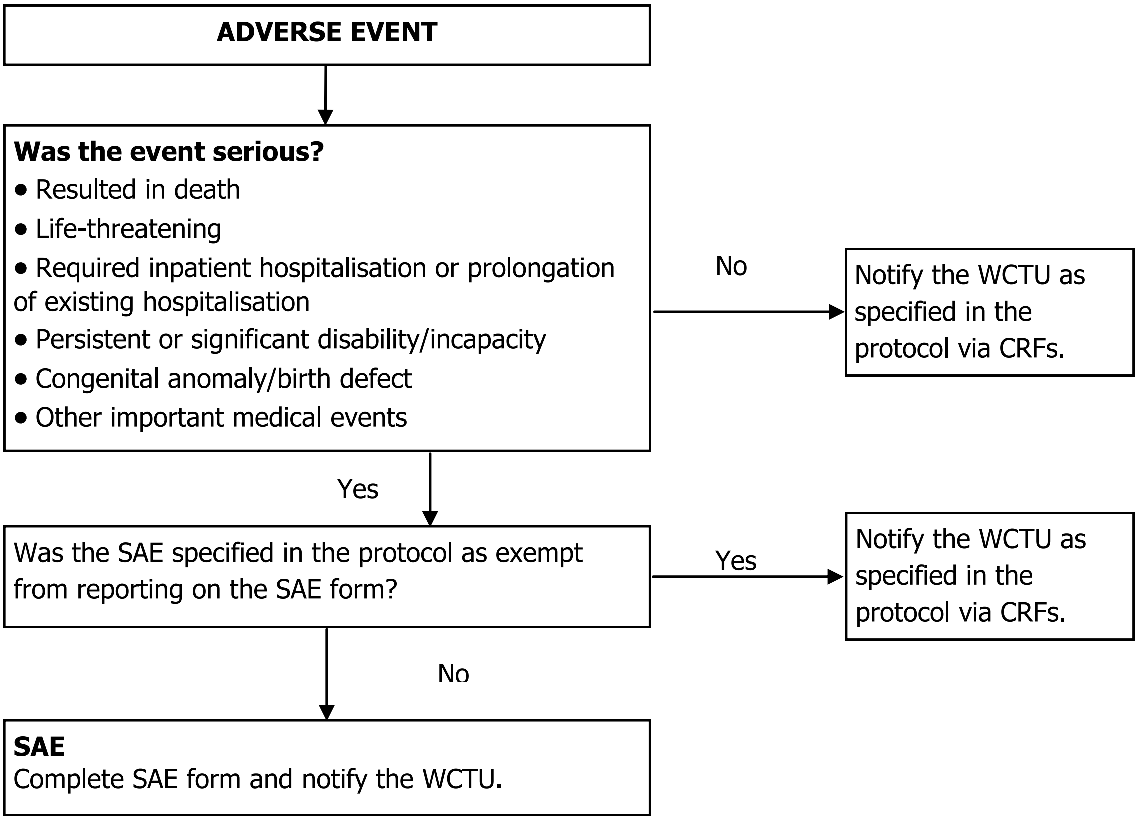

CRF Case Report Form
SAE Serious Adverse Event
WCTU Wales Cancer Trials Unit

11.0 Trial conduct and monitoring

11.1 Monitoring and quality assurance

Investigators agree to allow trial related monitoring, including audits and regulatory inspections, by providing direct access to source data/documents as required. Patient consent for this will be obtained.

The conduct of the trial is being overseen by the following committees:

1. An independent Trial Steering Committee (TSC) should meet at least annually and consider each report of the Independent Data Monitoring Committee (IDMC), as well as results of other trials and new information which has arisen, and recommend appropriate action.

2. The Trial Management Group (TMG) should meet at least once every six months to advise in the promotion and running of the trial. The TMG members include active trial investigators, WCTU representatives, Chief Investigators and specialist advisors (e.g. Health Services research and qualitative research advisors, Statistician, consumer representative and oncology representative). Minutes of the TMG meetings will be forwarded to the sponsor as the decisions based on these meetings may impact on the sponsorship arrangements.

11.2 Central monitoring

The top copy of each completed CRF should be returned to the WCTU for data entry within four weeks of the visit. The remaining copy is to be retained at the local centre.

CRF pages and data received by the WCTU from participating trial centres will be checked for missing, illegible or unusual values (range checks) and consistency over time.

If missing or questionable data are identified, a data query will be raised on a data clarification form. The data clarification form will be sent to the relevant participating site. The site shall be requested to answer the data query or correct data on the data clarification form. The case report form pages should not be altered.

All answered data queries and corrections should be signed off and dated by a delegated member of staff at the relevant participating site. The completed data clarification form should be returned to the WCTU and a copy retained at the site along with the participants case report forms.

The WCTU shall send reminders for any overdue data. It is a centre's responsibility to submit complete and accurate data in timely manner.

The University of Nottingham reserves the right to a Sponsor's designee having access to trial data for purposes of review at any time.

11.3 Participant withdrawal

In consenting to the trial, participants are consenting to NMES, trial follow up and data collection. If a participant wishes to withdraw from trial treatment, participating centres should nevertheless explain the importance of remaining on trial follow up for the purposes of data capture only. If the participant explicitly states their wish not to contribute further data to the trial, the WCTU should be informed. A completed withdrawal CRF should be faxed to the WCTU with the hard copy to follow soon after. Participants do not have to give a reason for their withdrawal but centres should make a reasonable attempt to find out why.

Participants should be withdrawn from the trial if they experience any of the following:

- Inability to tolerate electrodes and/or NMES
- Cessation of chemotherapy after 1 or 2 cycles

- Development of a complication that would preclude the use of NMES

Following withdrawal, no further clinical or non-clinical interventions or procedures would be carried out on the participant under the study protocol. No new samples or personal data would be collected. Data already collected in relation to the participant may be retained and used for the purposes for which consent has already been given, provided they are effectively anonymised and no longer identifiable to the research team or any other persons to whom access will be given.

11.4 Trial closure

The treatment phase will be followed by a non-interventional follow up period which will continue for 8 weeks after the last participant completes protocol treatment.

For the purposes of the Research Ethics Committee approval, the study end date is deemed to be the date of last data capture. The expected end date is 01/02/2012.

12.0 Statistical considerations

12.1 Randomisation

Randomisation will take place centrally after confirmation of eligibility by a telephone call to the WCTU. Participants will be randomised using balanced blocks. This will ensure balanced treatment allocation by a number of clinically important stratification factors. Randomisation will have an allocation ratio of 2:1.

12.2 Outcome measures

12.2.1 Primary outcome measure

Adherence to NMES: Proportion of patients completing a pre-determined level of compliance to the recommended NMES programme (NMES for 30min three times a week) measured using a self-report daily diary.

To comply, participants must complete three sessions during each week of chemotherapy, sessions of use must be for the full 30 minutes for each individual day, use beyond 30 minutes in one session or beyond the three required sessions per week can not be carried over and missing diary data will be treated as non-compliance

12.2.2 Secondary outcome measures

Safety of NMES: toxicities will be assessed according to the NCI Common Terminology Criteria for Adverse Events (NCI CTCAE v3.0). Serious adverse events will be monitored in "real-time".

Quadriceps muscle strength: peak torque (kg) measured using a portable Manual Muscle Tester dynamometer (Lafayette Instruments, USA).
Body composition: lean tissue mass of the body and thighs (kg) assessed by low-dose dual energy x-ray absorptiometry (DEXA).
Physical activity level: mean daily step count assessed by a small, lightweight activPAL™ accelerometer worn on the thigh for one week.
Response to chemotherapy: overall objective clinical response categorised using Response Evaluation Criteria In Solid Tumours, documented from patients' medical reports.
Nutritional intake: mean daily energy (kJ/d) and protein (g/d) intake estimated from a 3-day food and drink diary.
Fatigue: Multidimensional Fatigue Inventory.
Quality of life: the EORTC-C30 and the lung cancer specific module LC-13.
Experience of NMES - semi-structured interview exploring patient experiences of completing NMES during chemotherapy.

12.3 Sample size calculation

This trial is powered as a Fleming's single stage phase II trial of the intervention arm. The main endpoint is the proportion of patients completing a pre-determined level of compliance to the recommended NMES programme (NMES for 30min three times a week) measured using a self-report daily diary. Following advice from the cachexia subgroup of the NCRI Palliative Care Clinical Studies Group we have determined that 80% of patients should adhere to this minimum level in order to warrant a future phase III study. Conversely, if ≤50% adhered to this minimum level, this would give grounds to reject further study. Using a Fleming's one stage method, 20 patients will be required to distinguish between these rates (90% power, $p=0.05$) and if ≥15 out of 20 patients adhere, this would warrant further investigation.[17]
We have included a randomised control arm into this trial design in order to allow comparison of some secondary end points. Originally the trial started by randomising with a 1:1 allocation; however this was changed to a 2:1 in favour of the NMES arm allocation part way through the trial in order to support recruitment. As the study analysis is based on a within-intervention arm analysis, the statistical integrity of the study is not affected. A local audit suggests about 25% of patients drop out before reaching 3 cycles. We

require 20 patients in the treatment arm to be available for analysis; therefore we estimate that 26 patients need to be recruited to this arm. At the time of the change in the randomisation allocation ratio this meant that 47 patients would need to be recruited in total (26 to the treatment arm and 21 to the control arm).

12.4 Interim analyses and the role of the Independent Data Monitoring Committee

Interim analyses of the accumulating data will be performed at regular intervals for review by the Independent Data Monitoring Committee (IDMC). These analyses will be performed by the WCTU.

The IDMC will review the activity, safety and accrual data and, with consideration of any results from any relevant external trials, make a recommendation to the Independent Trial Steering Committee (TSC) on whether the trial should continue recruitment or close to recruitment. While the trial is recruiting, only the appropriate staff at the WCTU and the IDMC will see the results of the interim analyses.

12.5 Statistical analyses

A full statistical analysis plan will be developed before the first interim analysis of the trial data.

For the primary endpoint, the proportion (and 90% confidence interval) of patients completing NMES for 30min three times a week will be reported.

For the secondary (intention to treat) analyses, change in leg muscle strength from baseline will be compared between trial arms at 9, 17 and 20 weeks.

Descriptive statistics will then be presented for the following measures at 9, 17 and 20 weeks:

- Change in QoL score from baseline
- Change in body composition from baseline
- Change in nutritional intake from baseline
- Change in fatigue from baseline

Response to chemotherapy at 9 and 12 weeks will be described in each trial arm.

12.6 Subgroup analyses

No formal subgroup analyses are planned. Exploratory analyses may be conducted to aid hypothesis generation if a phase III is developed

13.0 Sub-studies

Qualitative interview study.

Background:
The importance of seeking the user perspective is becoming increasingly acknowledged and such perspectives need to be elicited in an ethically sensitive manner [18]. Several studies have explored the lived experience of cancer with some studies exploring the effects of specific interventions designed to enhance quality of life. [19, 20] To date, no study has investigated the perspectives of patients receiving NMES during chemotherapy.

Aim:
To add to the information available on patients' perspectives of NMES and to generate rich data to appreciate the meanings, benefits or otherwise of NMES in the context of patients' daily lives.

Methods:
Design:
A qualitative interview approach using Interpretative Phenomenological Analysis (IPA) [21] to explore patients' experience. Audiotaped and transcribed semi-structured interviews will record explorative discussions of individual patients' experience.

Participants:
Purposive sampling will be used. As the focus of IPA is on the lived experience of the individual, studies using this methodology are usually conducted using small patient numbers per homogeneous group This study will define the research group as patients taking part in the trial. [21].It is assumed that thematic saturation will be reached after 6-10 [22] interviews. The research physiotherapist will approach patients at week 9 or 12 assessments and invite them to take part in this sub-study. All patients are eligible for this sub-study until the qualitative researchers judge that thematic saturation has been reached when the sub-study will be closed. The patient contact details will then be passed to the qualitative researcher in order to arrange interview appointments. Interested patients will be consented and interviewed in clinic or their own home.

Ethical Considerations:
This client group represents a vulnerable population, who may have issues related to intrinsic, extrinsic and relational vulnerability. Ethical considerations for this aspect of the study are as follows:

1. Environmental Safety – Each client will not be interviewed for more than 30-40 minutes to minimise risk of tiring or compromising the participant. Support is available for each participant by referral to professional support (locally or nationally, e.g. Macmillan helpline, as per the Patient Information Sheet) should any distress arise as a result of the interview.
2. Information Management – Each participant will receive both written and verbal information about the study, in order to give informed consent. Signed consent forms will be stored in the patient notes and site master files. All fieldnotes and tapes will be stored in a secure location i.e. password protected computer or locked filing cabinet, viewed only by the researcher. Tapes will be destroyed after transcription.
3. Risk of patient distress – There is an awareness of the vulnerable nature of this group and the research group include a physiotherapist and psychologist with experience in palliative and supportive care.
4. If a clinical issue emerges, this will be referred to the participant's clinical team, with their permission.

Data Collection Methods:
The interview schedule (appendix A) is developed from available literature [18-20, 23, 24] and encourages exploration and discussion of the following areas:

- perceived experiences of NMES usage
- what is perceived to be important to patients about using NMES in the context of their illness and a clinical trial treatment
- the role of specific and non-specific effects of NMES treatment in impacting on perceptions of wellbeing and function
- factors affecting compliance and non-compliance

- other factors that may influence perspectives, or pertinent topics raised by the interviewees.

Data Collection Procedure:
Participants will be interviewed for a maximum duration of 30–40 minutes. These interviews will be digitally audio- taped, and the researcher will take notes to inform reflexive reflection. All interviews will be audio-taped and transcribed verbatim and a sample of transcripts will be reviewed by the qualitative collaborator to cross-check that appropriate themes are being identified within the analysis.

Analysis of Data:
The semi-structured interviews will be transcribed verbatim and examined for emergent themes using Interpretative Phenomenological Analysis (IPA) to indicate common themes that arise from experience of using NMES.

IPA is based on an idiographic approach beginning with a single case as a basis to develop more general categories developed in a detailed case-by-case analysis. The transcripts will be systematically analysed in several stages:

- Preliminary reading. The first transcript is read line-by-line and annotated with initial comments.
- Early analysis. Initial comments are grouped into themes.
- Higher level abstraction. Connections between themes are developed until an organised master list and thematic account of the case is achieved.
- Subsequent transcripts. New themes are tested against the previous transcripts as non-recurring themes are tested against following transcripts. Connections across cases are noted to identify a set of superordinate themes for the group.

Results will be analysed for consistent themes. A coding framework for emergent themes will be developed and then validated and compared.

Reflexivity:
In developing the semi-structured interview schedule, questions were developed to discuss the salient areas, without being leading. It is difficult however, in the context of an interview where inevitably the interpretations and understanding of the researcher can shape responses and influence the direction of the conversation[25]. Every effort in question design and implementation will be made to avoid leading the participants in this way. To address the issue of bias and assumptions within analysis, a sample of transcripts with identified themes will be reviewed by Dr Annmarie Nelson to ensure that themes are being identified appropriately. At the midpoint of analysis Dr Nelson will be invited to suggest revisions, which will be incorporated into the overall themes reached. This will validate the themes generated and reduce bias.

14.0 Publication policy

Data from all centres will be analysed together and published as soon as possible. Individual participating PIs may not publish data concerning their participants that are directly relevant to questions posed by the trial until the TMG has published its report. The TMG will form the basis of the writing committee and advise on the nature of publications, subject to sponsor requirements.

The main publication should include the Chief Investigator and Research Physiotherapist to represent the University of Nottingham and Gareth Griffiths and Anthony Byrne to represent Cardiff University/WCTU.

A publication arising from the qualitative sub-study should include the Chief Investigator, Research Physiotherapist to represent the University of Nottingham and Gareth Griffiths and Anthony Byrne to represent Cardiff University/WCTU as well as Annmarie Nelson and the qualitative researcher.

15.0 Informed consent, ethical and regulatory considerations

15.1 Ethical and other issues

This clinical trial protocol will be submitted to a Research Ethics Committee (REC) that is legally "recognised" by the United Kingdom Ethics Committee Authority for review and approval. The approval of the REC must be obtained before the start of a clinical trial or any trial procedures are conducted. The Local Research Ethics Committee (LREC) must also approve each institution, through the site specific assessment (SSA) process, before patients are recruited at that centre.

All substantial amendments to this trial protocol must be approved by the Main REC responsible for the study, before the implementation of the amendments. Minor amendments will not require prior approval by the Main REC.

If the trial is stopped due to adverse events it will not be recommenced without reference to the Main REC responsible for the study.

The Main REC will be notified within 90 days of trial completion. If the trial is terminated early, the Main REC will be notified of this within 15 days.

A summary of the clinical trial report will be submitted to the Main REC responsible for the study within one year of the completion of the last participant's final follow up procedure.

The patient's consent to participate in the trial should be obtained after a full explanation has been given of the treatment options, including the conventional and generally accepted methods of treatment. All patients must be informed of the aims of the study, the possible adverse events, the procedures and possible hazards to which they may be exposed. They will be informed of the strict confidentiality of their patient data, but that their medical records may be reviewed for trial purposes by authorised individuals other than their treating physician.

Patient's consent will be sought to notify their GP of their involvement in the trial. Patients should be given sufficient time after being given the trial Participant Information Sheet to consider and discuss participation in the trial with friends and family. A contact number should be given to the patient should they wish to discuss any aspect of the trial. Following this, the randomising investigator should determine that the patient is fully informed of the trial and their participation, in accordance with the principles of ICH-GCP. Patients should always be asked to sign a consent form. One copy should be given to the participant but the original copy should be kept in the study site file and a further copy should be kept with participant's hospital notes.

The right of the participant to refuse to participate in the trial without giving reasons must be respected. After the patient has entered the trial, the investigator must remain free to give alternative treatment to that specified in the protocol, at any stage, if he/she feels it to be in the best interest of the participant. However, the reason for doing so should be recorded and the participant will remain within the trial for the purpose of follow up and data analysis according to the treatment option to which he/she has been allocated. Similarly, the participant must remain free to withdraw at any time from the protocol treatment without giving reasons and without prejudicing his/her further treatment.

This is a randomised trial, therefore neither the participants nor their physicians will be able to choose the patient's treatment. Treatment will be allocated randomly using a computer-based algorithm. This is to ensure that the groups of participants receiving each of the different treatments are similar.

15.2 Research Governance approval

This trial protocol will be submitted through the Research Governance process of the host care organisation for review and approval. The Research Governance approval of the host care organisation must be obtained before the start of the trial within that host care organisation.

15.3 Sponsorship

NMES trial is being sponsored by Nottingham University. Nottingham University shall be responsible for ensuring that the clinical study is performed in accordance with the following:

- ICH Harmonised Tripartite Guideline for Good Clinical Practice
- Declaration of Helsinki 1996, latest amendment 2008
- Research Governance Framework for Health and Social Care 2nd edition 2005

Nottingham University has delegated the following responsibilities to the WCTU:

Obtaining appropriate ethics committee opinion:

- Selection of investigators and ensuring each centre has full trial documentation
- Ensuring an appropriate ethics committee opinion has been sought and any amendments approved
- Keeping records of all adverse events reported by investigators
- Having quality control systems in place to ensure that the study is conducted according to GCP at all participating centres
- Monitoring of the study.

The following responsibilities are delegated to the individual participating centres:

- Have in place arrangements to adhere to GCP
- Keep a copy of all essential documents (as defined in ICH-GCP) and ensure appropriate archiving and destruction once the study has ended.
- Take appropriate urgent safety measures.

15.4 Indemnity

Insurance and indemnity for trial participants and trial staff is covered within the NHS Indemnity Arrangements for clinical negligence claims in the NHS, issued under cover of HSG (96)48. There are no special compensation arrangements, but trial participants may have recourse through the NHS complaints procedures.

The University of Nottingham has taken out an insurance policy to provide indemnity in the event of a successful litigious claim for proven non-negligent harm.

15.5 Data protection

The WCTU will act to preserve patient confidentiality and will not disclose or reproduce any information by which participants could be identified (except where participants are registered with the National Health Service Information Centre (formerly the Office for National Statistics) or traced via the NHS Central Register, which requires separate consent). Data will be stored in a secure manner and our trials are registered in accordance with the Data Protection Act 1998.

15.6 Finance

The NMES trial is being funded by the NCRI Supportive and Palliative Care (SuPaC) Research Collaborative, on behalf of the National Cancer Research Institute and thus is part of the NCRN/NCRI portfolio of clinical trials.

A subcontact has been signed between the sponsor, the University of Nottingham, and the WCTU host organisation, Cardiff University, outlining work agreements for this trial.

16.0 Trial committees and trial management arrangements

The Trial Management Group (TMG) coordinates and manages the trial's day-to-day activities. The TMG is comprised of the Chief Investigator, WCTU representatives, active Investigators and specialist advisors (e.g. Health Services research and qualitative research advisors, consumer representative and oncology representative).

The data will be reviewed (approximately six monthly) by an Independent Data Monitoring Committee (IDMC), consisting of at least two Clinicians (not entering patients into the trial) and an independent Statistician. The IDMC will be asked to recommend whether the accumulated data from the trial, together with results from other relevant trials, justifies continuing recruitment of further patients. A decision to discontinue recruitment, in all patients or in selected subgroups, will be made only if the result is likely to convince a broad range of Clinicians including PIs in the trial and the general clinical community. If a decision is made to continue, the IDMC will advise on the frequency of future reviews of the data on the basis of accrual and event rates. The IDMC will make confidential recommendations to the Trial Steering Committee (TSC).

The role of the TSC is to act on behalf of the sponsor and funder, to provide overall supervision for the trial, to ensure that it is conducted in accordance with GCP, and to provide advice through its independent chairman. The TSC will review the recommendations from the IDMC and will decide on continuing or stopping the trial, or modifying the protocol.

17.0 References

1. National Collaborating Centre for Acute Care, February 2005. Diagnosis and treatment of lung cancer. National Collaborating Centre for Acute Care, London. Available from www.rcseng.ac.uk
2. Ancoli-Israel S, Moore PJ, Jones V. The relationship between fatigue and sleep in cancer patients: a review. *Eur J Cancer Care* 2001;10:245–255.
3. St-Pierre DM, Kreisman H, Kasymjanova G et al. Quality of life and survival in patients receiving chemotherapy for advanced non-small cell lung cancer (NSCLC). *JCO* 2007;25(18s):19649.
4. Kasymjanova G, Kreisman H, Pepe C et al. Effect of chemotherapy on exercise capacity in patients with advanced non-small cell lung cancer (NSCLC). *JCO* 2007;25(18s):9115.
5. Conn VS, Hafdahl AR, Porock DC et al. A meta-anlaysis of exercise interventions among people treated for cancer. *Supportive Care in Cancer*. 2006;14:699–712.
6. Oldervoll LM, Loge JH, Paltiel H et al. The effect of a physical exercise program in palliative care: a phase II study. *J Pain Symptom Manage* .2006;31:421–430.
7. Stevinson C and Fox KR. Feasibility of an exercise rehabilitation programme for cancer patients. *European Journal of Cancer* 2006;15:386–396.
8. Neder JA, Sword D, Ward SA et al: Home based neuromuscular electrical stimulation as a new rehabilitative strategy for severely disabled patients with chronic obstructive pulmonary disease (COPD). *Thorax* 2002;57:333–337.
9. Quittan M, Wiesinger GF, Strum B et al: Improvement of thigh muscles by neuromuscular electrical stimulation in patients with refractory heart failure: a single randomised controlled trial. *Am J Phys Med Rehabil* 2001;80:206–214.
10. Martin HJ, Yule V, Syddall HE et al. Is hand-held dynamometry useful for the measurement of quadriceps strength in older people? A comparison with the gold standard biodex dynamometry. *J Gerontol* 2006;52:154–159.
11. O'Shea SD, Taylor NF, Paratz JD. Measuring muscle strength for people with chronic obstructive pulmonary disease: retest reliability of hand-held dynamometry. *Arch Phys Med Rehabil* 2007;88:32–36.
12. Ryan CG, Grant PM. The validity and reliability of a novel activity monitor as a measure of walking. *Br J Sports Med* 2006;40:779–784.
13. Therasse P, Arbuck SG, Eisenhauer EA et al. New guidelines to evaluate the response to treatment in solid tumors. European Organization for Research and Treatment of Cancer, National Cancer Institute of the United States, National Cancer Institute of Canada. *J Natl Cancer Inst* 2000;92:205-216.
14. Smets EMA, Garssen B, Bonke B et al. The multidimensional fatigue inventory (MFI) psychometric qualities of an instrument to assess fatigue. *J Psychosomatic Research* 1995;39:315–325.
15. Smith JA and Osborn M (2003) Interpretative phenomenological analysis. In JA Smith (ed) *Qualitative Psychology: A Practical Guide to Methods.* London: Sage.
16. Bax L, Staes F, Verhagen A. Does neuromuscular electrical stimulation strengthen the quadriceps femoris? *Sports Medicine* 2005;35(3):191–212.
17. Fleming TR. One-sample multiple testing procedure for phase II clinical trials. *Biometrics* 1982;38:143–151.
18. 18. Gambles M, Crooke M, Wilkinson S. Evaluation of a hospice based reflexology service: a qualitative audit of patient perceptions. Eur J Oncol Nurs 2002;6:37–44.
19. Korstjens L, Mesters I, Gijsen B, van den Borne, Cancer patients' view on rehabilitation and quality of life: a programme audit. Eur J Cancer Care 2008;17:290–7.
20. Bertero C, Vanhanen M, Appelin G. Receiving a diagnosis of inoperable lung cancer: patients' perspectives of how it affects their life situation and quality of life. Acta Oncol 2008;47:862–9.
21. Smith JA. Reflecting on the development of interpretative phenomenological analysis and its contribution to qualitative psychology. Qualitative Research in Psychology 2004;1:39-54.
22. Turpin G, Barley V, Beail N et al. Standards for research projects and theses involving qualitative methods: suggested guidelines for trainees and courses. Clinical Psychol forum 1997;108:3-7.
23. Larkin M, Watts S, Clifton E. Giving voice and making sense in interpretative phenomenological analysis. Qualitative research in Psychology 2006;3:102-120.
24. Dean S, Smith JA, Weinman J et al. Managing time: an interpretative phenomenological analysis of patients' and physiotherapists' perceptions of adherence to therapeutic exercise for lower back pain. Disability and Rehab 2005;27:625-636.
25. Matterud K. Qualitative research: Standards, Challenges and Guidelines. Lancet 2001;358:483-488.

APPENDIX 1: Interview schedule

1. Could you tell me a little bit about your experience of using NMES. How have you found using the device?

2. What benefits do you see of using the device.

3. Are there any aspects of using the device that you found difficult

4. Did you manage to carry out the intervention when you were feeling particularly tired or unwell?

5. How important is your relationship with the team who are treating you?

(The therapeutic relationship has been identified as being potentially important as a factor valued by patients in the process of care, this question aims to facilitate exploration of this area with the patient.)

6. Has having NMES treatment had any effect on your life in terms of:
    - Quality of life
    - Coping ability
    - How you feel in yourself
    - Any other reason

(This is an extensive area of exploration looking at effect or benefit. The aim is to ask this generally "has the NMES intervention had any effect on your life" and then extends this to the specific areas of interest indicated by the literature)

7. Do you think it is important that you are involved in the management of your condition?

8. Is there anything else you would like to tell me about your experience of using NMES that you feel is important?
